# The Role of Genetic and Epigenetic Regulation in Intestinal Fibrosis in Inflammatory Bowel Disease: A Descending Process or a Programmed Consequence?

**DOI:** 10.3390/genes14061167

**Published:** 2023-05-27

**Authors:** Sara Jarmakiewicz-Czaja, Aneta Sokal, Katarzyna Ferenc, Elżbieta Motyka, Kacper Helma, Rafał Filip

**Affiliations:** 1 Institute of Health Sciences, Medical College of Rzeszow University, 35-959 Rzeszow, Poland; 2 Institute of Medicine, Medical College of Rzeszow University, 35-959 Rzeszow, Poland; 3 Centre for Innovative Research in Medical and Natural Sciences, Medical College of Rzeszow University, 35-959 Rzeszow, Poland; 4 Department of Gastroenterology with IBD, Clinical Hospital No. 2 im. Św. Jadwigi Królowej, 35-301 Rzeszow, Poland

**Keywords:** Crohn’s disease, epigenetic factors, intestinal fibrosis, *NOD2*, *TGFβ*, ulcerative colitis

## Abstract

Inflammatory bowel diseases (IBDs) are a group of chronic diseases characterized by recurring periods of exacerbation and remission. Fibrosis of the intestine is one of the most common complications of IBD. Based on current analyses, it is evident that genetic factors and mechanisms, as well as epigenetic factors, play a role in the induction and progression of intestinal fibrosis in IBD. Key genetic factors and mechanisms that appear to be significant include *NOD2*, *TGF-β*, *TLRs*, *Il23R*, and *ATG16L1*. Deoxyribonucleic acid (DNA) methylation, histone modification, and ribonucleic acid (RNA) interference are the primary epigenetic mechanisms. Genetic and epigenetic mechanisms, which seem to be important in the pathophysiology and progression of IBD, may potentially be used in targeted therapy in the future. Therefore, the aim of this study was to gather and discuss selected mechanisms and genetic factors, as well as epigenetic factors.

## 1. Introduction

The process of fibrosis is a consequence of recurrent and continuous wound damage and regeneration [1,2]. Intestinal fibrosis is one of the most common and serious complications of inflammatory bowel disease (IBD). It can occur in Crohn’s disease (CD) and ulcerative colitis (UC), while complications of CD, such as intestinal obstruction, perforation, and fistulas, are more common [3]. One of the factors that activate the process of intestinal fibrosis in IBD is chronic inflammation, resulting in an excessive accumulation of scar tissue on the intestinal wall. The process of fibrosis can lead to post-inflammatory narrowing of certain intestinal segments, resulting in altered structure and function. The clinical picture and the treatment of IBD in such cases become more complex [4]. Wang et al. indicated that in more than half of patients with CD, there might be post-complications associated with intestinal fibrosis, such as organ stenosis and/or penetration [5]. However, in UC, stricture formation can occur in up to about 10% of cases, but the percentage of colonic strictures is comparable in both CD and UC. In the course of UC, colonic fibrosis can cause significant clinical symptoms, such as intestinal motility disorders and rectal dysfunction [6]. Up to one-fifth of patients with IBD in Europe are estimated to develop intestinal strictures [7]. Approximately 40% of CD patients with localized inflammation in the ileum experience obstruction symptoms, and 70–80% require intestinal surgery within 20 years after the diagnosis of the disease [8].

**Figure 1:**
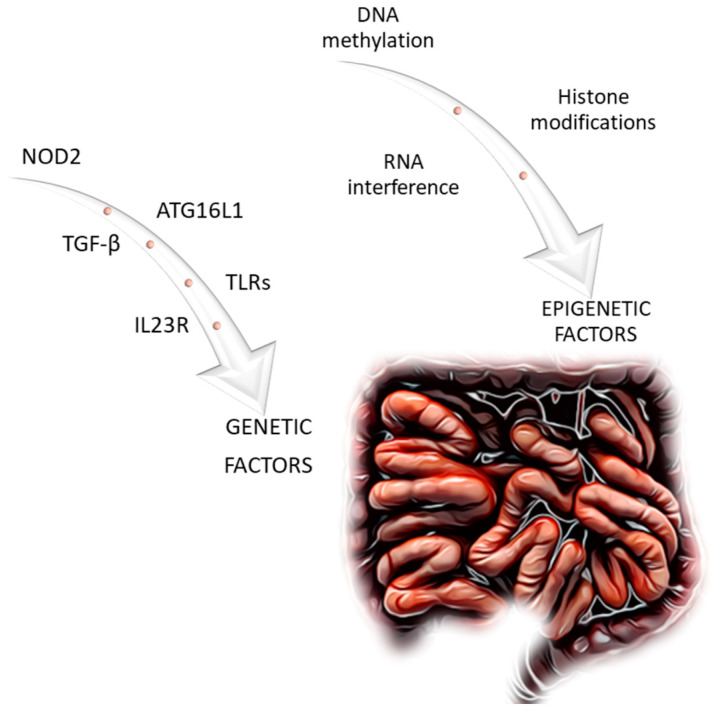
Genetic and epigenetic factors related to intestinal fibrosis in Crohn’s disease discussed in this article.

The pathophysiology of intestinal fibrosis is not fully understood. Chronic inflammation is considered the main factor. Furthermore, in recent years, researchers have identified genetic predisposition and epigenetic factors (Figure 1) that affect the development of intestinal fibrosis in IBD [9,10]. Li et al. indicate that these may include histone modifications, microRNA (miR) expression, or changes in deoxyribonucleic acid (DNA) methylation. CD and UC are polygenic diseases. There are genetic mutations whose presence increases the risk of IBD. A genome-wide association study (GWAS) identified more than 200 *loci* that are associated with IBD risk. However, a thorough analysis of the GWAS results does not fully determine the genomic basis that determines the CD phenotype. Genetic predisposition combined with epigenetic mechanisms that affect genes and key IBD pathways can lead/contribute to the development of intestinal fibrosis [10].

The factors linking IBD and intestinal fibrosis are not yet fully understood. The main purpose of this review is to update and collect information on the genetic and epigenetic predisposition to the appearance of intestinal fibrosis in IBD and to find information gaps that could lead researchers to design future studies.

## 2. Inflammatory Bowel Disease (IBD)

Inflammatory bowel disease is a chronic and recurrent inflammatory bowel disease that can be caused by multiple factors. The etiology of IBD has not been fully investigated, but many studies suggest that genetic, immunological, microbiological, and environmental factors and their interactions have a significant impact on the mechanism of formation and development of IBD. Studies show that in IBD, genetically predisposed individuals develop abnormalities in the immune system due to environmental factors [11,12,13]. The disease is characterized by a chronic course with alternating exacerbations and remissions of the symptoms of the disease. The severity and course of the disease are specific to each patient and depend on the location and size of the inflammation area [12]. The three main IBD subtypes are CD, UC, and microscopic colitis (MC). UC and CD are the most common and occur in both sexes regardless of age. Young people tend to be affected more frequently, but most patients are diagnosed with UC in their third or fourth decade of life, and when it comes to CD, they are diagnosed with it in their second or third decade of life [14,15]. MC is less common and affects older people. The median age of patients with MC at the time of diagnosis is older than 60, but cases of the disease have also been reported at ages below 45 and even in children [16].

The clinical picture, course, differentiation, prognosis, and treatment of these three subtypes of IBD differ. In CD, the inflammatory process begins in the mucosa but gradually involves all layers of the gastrointestinal wall, leading to its destruction and fibrosis and resulting in the formation of fistulas and strictures. In UC, the inflammatory process involves the mucosa and submucosa of the rectum or colon [14].

IBD is classified as a global disease. The incidence and prevalence of the disease depend on the geographic region and are closely related to the industrialization and urbanization of the world. Initially, the highest number of cases was recorded in the western part of Europe, North America, and Oceania, where the incidence increased rapidly with the development of the economy and medical care. It was not until the turn of the twentieth and twenty-first centuries that the incidence of IBD in these countries began to stabilize. Therefore, there is now an increase in the incidence of IBD in developing countries such as Asia, South America, and Africa, where Western lifestyles are beginning to permeate the landscape. The incidence of IBD in newly industrialized countries is much lower than in Western countries but is increasing rapidly. Researchers suggest that with the passage of time and development in these countries, the incidence of IBD may reach levels similar to those currently found in Europe. The global increase in the prevalence of IBD is of great concern for both patients and countries alike, as the disease significantly affects the quality of life and treatment costs, and the complexity of the disease poses a challenge to the healthcare system [17,18].

Chronic inflammation in IBD can lead to the serious complication of intestinal fibrosis, which can predispose to intestinal strictures and obstructions. In such cases, the risk of mortality increases, and the duration of hospitalization is also prolonged [19,20,21].

## 3. Intestinal Fibrosis

Fibrosis is a progressive complication of most chronic inflammatory diseases that develops in response to tissue damage. Many organs can be affected by it, which can often lead to organ failure or even death [22]. Intestinal fibrosis is a common complication of CD and UC. It can lead to the formation of strictures and subsequent intestinal obstruction that decreases the quality of life of patients. It usually requires surgical intervention [23]. Intestinal fibrosis can be considered an extreme healing response in IBD. It is described as a degeneration of connective tissue, in which functional tissue is replaced by excessive accumulation of fibrillar collagen-rich extracellular matrix (ECM). It develops after recurring stimulation of intestinal tissue by chronic inflammation [1].

The accumulation of ECM is a natural part of tissue repair and recovery after an injury in all organs [24]. When tissues are damaged, local tissue fibroblasts are activated and begin the wound-healing process. They increase the secretion of inflammatory mediators, synthesize ECM components, and increase their contractility. Under normal conditions, with minor and non-chronic tissue damage, the healing process causes a transient increase in the deposition of components of ECM and leads to the restoration of the functional tissue architecture [25]. The ECM stabilizes mechanically damaged tissue and acts as a cell instructor. At the beginning of the wound-healing process, it immobilizes growth factors such as transforming growth factor beta (TGF-β), platelet-derived growth factor (PDGF), vascular endothelial growth factor, and fibroblast growth factor. Then it serves as a scaffold to transport fibroblasts, immune cells, and endothelial cells to damaged tissue [24]. When the injury is severe or repetitive, with persistent inflammation, such as CD, it can lead to excessive accumulation of ECM, resulting in progressive fibrosis and hardening and scarring of intestinal tissues [1,25]. Eventually, it can affect organ function and cause lasting damage [6,25]. It seems that inflammation may be a key factor in determining whether wound healing is proceeding properly, but it is worth noting that suppression of inflammation alone does not prevent the development of intestinal fibrosis [25].

Many cell types in the intestinal tract can become activated ECM-producing myofibroblasts. These can be cells derived from resident mesenchymal cells but also from epithelial cells, endothelial cells, stellate cells, pericytes, or bone marrow stem cells [26]. Resident fibroblasts and pericytes are the main precursors of scar-forming myofibroblasts [27]. Intestinal mesenchymal cells such as fibroblasts, myofibroblasts, and smooth muscle cells (SMCs) can differentiate from each other in the inflamed gut and are considered primarily responsible for ECM production in the intestine [28,29]. Myofibroblasts have an intermediate phenotype between fibroblasts and SMCs. These cells exhibit the ability to contract and migrate within tissues and are believed to be one of the main cells that overproduce ECM under the influence of various factors [20,30]. Fibroblasts and myofibroblasts in IBD can be exposed to multiple cytokines released by various cell types [6]. Chronic inflammation-induced cytokine release can trigger and directly promote the development of intestinal fibrosis; however, it is unclear whether the neutralization of a single cytokine or a combination of multiple cytokines will effectively prevent or reverse intestinal fibrosis [31]. Epithelial cells, endothelial cells, and fibrocytes contribute to fibrogenesis by producing important fibrogenic cytokines and promoting intercellular communication [27].

The development of fibrosis can also be influenced by the balance between the synthesis and degradation of ECM. Matrix metalloproteinases (MMPs) are the main proteolytic enzymes that degrade ECM. The enzyme activity of MMPs is controlled by tissue inhibitors of metalloproteinases (TIMPs). TIMPs can inhibit ECM proteolysis directly or indirectly, and an imbalance between TIMPs and MMPs can cause excessive accumulation and fibrosis of ECM in the intestine [6,32].

Macrophages are crucial for the wound-healing process. Various subsets of macrophages produce profibrotic mediators or enzymes that degrade ECM that contribute to the maintenance of ECM homeostasis [1]. They also phagocytize debris and produce chemokines, PDGF, TGF-β, and vascular endothelial growth factor. They can attract fibroblasts, pericytes, endothelial, and smooth muscle cells that participate in the proliferative phase of wound healing [24]. Disruption of macrophage recruitment and activation can lead to incomplete tissue regeneration by attenuating the early inflammatory response, reducing wound debridement, and prolonging exposure to pro-inflammatory stimuli. Eventually, it may lead to the development of pathological fibrosis or scarring [33].

Fibrosis is associated with recurrent episodes of inflammation and wound healing; however, current anti-inflammatory IBD therapies do not appear to reduce the rate of stenosis, suggesting that inflammation-independent mechanisms also contribute to intestinal fibrogenesis. Mitochondrial dysfunction may play an important role in both the onset and recurrence of IBD [34]. Most cellular functions and maintenance of the epithelial barrier are energy-dependent, so it appears that mitochondrial dysfunction may also be related to intestinal fibrosis. Fibrosis has been shown to be associated with impaired mitochondrial respiratory chain in human lung biopsies [35]. In mice, protection against oxidative stress and mitochondrial dysfunction delays hepatic fibrosis [1]. Data on metabolic changes that occur during intestinal fibrosis are limited and include changes in glycolysis, fatty acid oxidation, and glutamine metabolism [1]. Interference with these pathways may potentially be part of fibrosis therapy in the future.

## 4. Genetic Factors and Mechanism of Intestinal Fibrosis in Inflammatory Bowel Disease

It is likely that variants in genes that encode pro- and anti-inflammatory cytokines, immunoregulatory proteins, and fibrinogen factors may influence fibrosis in IBD. Among genetic factors, the influence of the nucleotide-binding oligomerization domain-containing gene 2 (*NOD2*), the autophagy-related 16-like 1 gene (*ATG16L1*), the fibrogenic molecule that transforms TGF-β, and various variants of toll-like receptors (TLRs) (especially TL4) is prominent [36].

### 4.1. NOD2 (Nucleotide-Binding Oligomerization Domain-Containing 2)

The *NOD2* gene located at the IBD1 locus on chromosome *16q12* encodes *CARD1* and can contribute to transcriptional and translational changes. The *NOD2* protein is a cytoplasmic receptor that plays an important role in activating the innate immune system in the development of CD [37,38]. Cells carrying *NOD2* variants are associated with the fibrostenosis phenotype of CD and contribute to increased *TGF-β1* and collagen [39]. Patients with CD have an increased number of circulating fibrocytes, and these are strongly correlated with fibrostenotic disease in CD [40]. Although there are many reports on the association of *NOD2* with fibrostenotic disease, there is no consensus on its association with the localization of the disease in the intestine [37]. The results of a large multicenter study published in 2015 suggest that disease localization is partially genetically determined and is a major factor in changes in disease behavior over time [41]. In addition, autophagy is an intracellular degradation pathway in which cytoplasmic cargo is absorbed into a double membrane vesicle and delivered to the lysosome [38,42].

### 4.2. TGF-β (Transforming Growth Factor β)

TGF-β belongs to the TGF-β superfamily of ligands and is responsible for cell migrations and adhesion, growth, differentiation, and apoptosis, among other things. The deregulation of TGF-β expression and function is closely associated with the development of fibrosis [43]. The findings indicate elevated TGF-β levels in the mucosa of CD patients [44]. According to Chen et al., TGF-β1, as well as collagen 1α1, are the main drivers of fibrosis in the intestine. This occurs through an ER stress response in the supramembranous myofibroblasts of the ileum. DNA (cytosine-5)-methyltransferase 1 (DNMT1) dependent silencing of miR-199a-5p results in increased expression of TGF-β1 and collagen 1α1 in these cells [45]. It is also significant that TGF beta, in addition to being one of the genetic factors, can also influence epigenetic mechanisms [46,47,48].

### 4.3. TLRs (Toll-like Receptors)

Toll-like receptors (TLRs) are responsible for the increased expression of many inflammation-related genes that play an important protective role against infection. TLRs belong to the family of pattern recognition receptors (PRRs), which are responsible, among others, for the recognition of pathogens by the extracellular matrix [49,50]. In an animal model study, TLR4 deficiency has been shown to be associated with reduced colitis and macrophage infiltration into the colon, which has a direct impact on reduced collagen deposition and fibrosis development. Thus, TLR4 plays the role of a mediator in inflammation and fibrosis by regulating the expression of cytokines in intestinal macrophages and myofibroblasts [51].

### 4.4. Il23R (Interleukin 23 Receptor)

Interleukin 23 (IL23) is a pro-inflammatory cytokine with pleiotropic effects. Sewell and Kaser concluded that the importance of IL23 in the pathogenesis of autoimmune diseases, including CD and UC, is undisputed [52]. Studies indicate that variants of the IL23 receptor gene are associated with fibrosis in CD, specifically the TT genotype of the IL23R *rs1004819* variant associated with ileal CD and stricture [53].

### 4.5. ATG16L1 (Autophagy-Related 16-like 1)

The *ATG16L1* gene is located on chromosome 2, encodes a protein necessary for autophagy, and is responsible for the formation of the autophagosome [54]. Defects in the *ATG16L1* gene are closely associated with type 10 inflammatory bowel disease and promote fibrosis [55,56]. An alteration in the *ATG16L1* gene alters the process of autophagy, and persistent cells and bacteria can trigger an inappropriate response leading to inflammation [54]. Current research results indicate that mutation of genes in the autophagy pathway, including *ATG16L1*, *NOD2*, *LRRK2* (leucine-rich repeat kinase 2), *IRGM*, *ULK1* (Unc-51-like autophagy-activating kinase 1), increases the risk of developing severe fibrous CD [57].

## 5. Epigenetic Factors

Epigenetics is defined as any possible mechanism that ultimately leads to potentially heritable changes in the cell phenotype without modifying the underlying DNA sequence [58]. Epigenetic mechanisms regulate many different physiological and pathophysiological processes by influencing gene expression [59]. Proper epigenetic regulation is of significant importance in the context of cell development and proliferation control, as well as in maintaining genome integrity and transcriptional regulation [60]. Epigenetic control of gene expression is regulated through intracellular mechanisms, including DNA methylation, histone modifications, and ribonucleic acid (RNA) regulation [61,62,63]. Current analyses indicate that epigenetic changes can be induced by environmental stimuli and, more importantly, can be reversible [64].

Oxidative stress is due to an imbalance between reactive oxygen species (ROS) and antioxidants, which play a role in the natural antioxidant defense of the body [65]. ROS can affect DNA mutations, and these changes can, in turn, hypermethylate DNA [66]. In addition, ROS can also influence histone modifications [67]. Nuclear factor E2-related factor 2 (*NRF2*) is highly sensitive to redox reactions and encodes the *NFE2L2* gene. Furthermore, it is an important regulator of phase II antioxidant enzymes [68]. Silencing *NRF2* induces oxidative stress by inhibiting antioxidant defense activity [69].

### 5.1. Deoxyribonucleic Acid (DNA) Methylation

DNA methylation is the most well-known epigenetic mechanism [70]. Its role is particularly important in the regulation of gene expression, which is crucial for normal human development [71]. As a result of DNA methylation, a covalent attachment of a methyl group (–CH3) to cytosine or adenine is observed, using the enzyme DNA methyltransferase (DNMT) and S-adenosylmethionine (SAM) as a donor of the methyl group [72]. The methylation process occurs mainly on cytosine–guanine (CpG) dinucleotides. The vast majority of CpGs are located on the so-called CpG islands, where they are highly concentrated [73]. DNA methylation is a dynamically controlled process that can be reversed through cell division, during which it loses methyl groups, or through demethylation, which involves enzymatic catalysis [74]. In recent years, changes in DNA methylation have been associated with disease processes that induce tissue fibrosis, such as in the lungs, liver, or intestines during IBD. Hypermethylation of specific genes and general changes in methylation have been observed in these organs [75,76,77].

Somineni et al. compared whole genome DNA methylation analyses in 164 pediatric patients with CD at diagnosis and 74 healthy controls. The patients were observed for 5 years. They found that CpG methylation was different between the study group and the control group. Furthermore, these changes were correlated with C-reactive protein (CRP). In patients with IBD, methylation changes were observed during the treatment of the inflammatory state. These modifications resembled the patterns of the control group. It is suggested that the changes in CpG methylation associated with CD are the result of inflammation rather than contributing to the development of the disease [78]. Howell et al. also found changes in methylation patterns between newly diagnosed pediatric patients with IBD and the control group. Furthermore, they demonstrated differences in the transcription of intestinal epithelial cells. This analysis suggests that modifications of the intestinal epithelium may be involved in the pathogenesis of IBD [79]. The study by Nimmo et al. reveals a global mechanism of methylation in CD, demonstrating altered regulation of significant immune defense mechanisms, including regulation of the Th17 pathway, IL-27, IL-19, and tumor necrosis factor alpha (TNFα) [80]. A study involving 149 patients with IBD identified multiple specific modifications in DNA methylation. The differentially methylated position (DMP) (*cg16176675*) was significantly hypermethylated in the CD and UC groups compared to the control group [81]. One of the studies aimed to use a DNA methylation chip to comprehensively assess changes in DNA methylation in the intestinal mucosa genome. The results of the analysis showed abnormalities in methylation in the *HLA-DRB1*, *YPEL5*, and *CBLB* genes in CD patients. The *MUC1* methylation error was negatively correlated with the active disease period [82]. Sadler et al. observed three genes in which differential DNA methylation in each part of the promoter was inversely correlated with gene expression in intestinal fibroblast fibrosis [83].

### 5.2. Histone Modifications

Eukaryotic cells store genetic information in DNA, which is packaged in a highly complex structure called chromatin [84]. Nucleosomes, the building blocks of chromatin, are made up of 147 base pairs of DNA wrapped around an octamer of proteins that contains 4 histones in 2 copies (*H2A*, *H2B*, *H3*, and *H4*) [85]. Histone modifications are important epigenetic mechanisms that regulate fundamental biochemical processes. This is achieved by modifying chromatin and gene expression [86]. There are various ways of histone modification, including methylation, acetylation, phosphorylation, ubiquitination, and ribosylation of adenosine-5’-diphosphate (ADP) [87,88].

Zhou et al. demonstrated that inhibition of histone 3 methyltransferase EZH2, specifically at the position of lysine 3 (*H3K27me3*), through processes such as methylation, 7e-methylation, and trimethylation, initiated the development of myeloid-derived suppressor cells (MDSC) [89]. As a result, favorable conditions for anti-inflammatory action appeared in IBD therapy [90]. Another analysis presented *H3K27me3* as a protein that could potentially control extracellular vesicles to regulate Th17 in patients with UC [91]. A study in mice with DSS-induced colitis investigated whether inhibition of histone methyltransferase *H4K20 (SETD8)* affects the progression of IBD in patients. The results showed that silencing *SETD8* regulates the expression of the p62 protein, thus blocking the inflammatory response of the colon. The authors suggest that *SETD8* may be used in the future as a potential therapy for patients with IBD [92]. Another study using *Lactobacillus casei probiotic* in mice with colitis investigated the effect of *L. casei* on the immune response and histone modifications. In this study, it was observed that after DSS administration, the level of *H3K9* acetylation decreased, while supplementation with *L. casei* restored its normal level in colon cells [93]. Li et al. demonstrated in their study that protein arginine methyltransferase (*PRMT*) plays a crucial role as an epigenetic modifier in the development of IBD. *PRMT* has been found to show strong expression in patients with IBD. Overexpression of this methyltransferase decreases when symptoms of colitis are alleviated. It appears that *PRMT* may act as a mediator and a marker of the inflammatory state of the colon [94]. Sadler et al. demonstrated that the expression of the *COL1A2* gene, through the selective action of cytokines on chromatin, probably plays a significant role in the endothelial–mesenchymal transition (EndoMT) and intestinal fibrosis [95]. Acetylation and histone deacetylation are associated with pulmonary fibrosis. It appears that the mechanism of this action involves H3 hypermethylation through increased expression of histone deacetylase [96]. A similar mechanism of action occurs in liver fibrosis [97]. Importantly, differentiation of H3 patterns has also been detected in patients with IBD [98].

### 5.3. Ribonucleic Acid (RNA) Interference

Some of the best-known noncoding RNA (ncRNA) molecules are microRNAs (miRNAs), which are 19–24 nucleotide ncRNAs. Other long RNAs (lncRNAs) are more than 200 nucleotides long [99]. RNA interference is a post-transcriptional gene silencing mechanism that regulates gene expression by RNA molecules [64]. It appears that miRNA-induced interference in gene expression participates in the epigenetic regulation of fibrosis [100]. Examination of many miRNAs has been conducted; however, two miRNAs are commonly cited as being clearly associated with the expression and fibrosis of transforming growth factor beta (TGF-β) [62]. Increased expression of miR-21 in muscle cells and myofibroblasts in CD patients leads to continuous activation of TGF-β1 signaling, resulting in excessive production of collagen and extracellular matrix, leading to fibrosis [101]. In fact, several studies have shown that the Wnt-β-catenin signaling pathway is activated in intestinal fibrosis. Activation of this pathway is necessary in the case of TGF—induced fibrosis induced by TGF-β [102].

Further investigation of epigenetics in the context of the pathophysiology of intestinal fibrosis requires additional studies with precise methodology and a broad perspective on all factors that influence epigenetic mechanisms.

Table 1 summarizes genetic and epigenetic factors and their role in fibrosis.

## 6. Treatment of Intestinal Fibrosis

### 6.1. Potential Therapeutic Factors

One of the most important prophylactic goals for patients with IBD is to prevent the appearance of intestinal fibrosis and subsequent strictures. Assessing the risk of complications in IBD could be helpful in the implementation of treatment and in eliminating potential epigenetic factors that could predispose to intestinal fibrosis [105]. Differential diagnosis should be made when choosing the appropriate treatment; however, the differentiation of fibrous and inflammatory strictures remains a challenge due to the fact that inflammatory factors and fibrosis often overlap [106]. 

Treatment of patients with IBD with comorbid intestinal fibrosis with stenosis is a multidisciplinary problem, as it is based on drug therapy, postoperative management, and endoscopic [107]. Due to the lack of targeted antifibrotic therapy in IBD, efforts are still being made to develop standards for the treatment of fibrotic intestinal stenosis in IBD [6,108]. Magnetic resonance imaging (MRI) is the preferred imaging technique to assess stenosis and patient response to the treatment used [21]. 

D’Haens et al. point out that, most likely, antifibrotic treatment alone is not enough and, consequently, anti-inflammatory treatment must also be introduced, so the authors conclude that the design of “combined interventions” studies should be considered [109]. Developing an appropriate and targeted treatment to reduce the risk of fibrosis or reverse its symptoms is hampered by the lack of markers and diagnostic methods for fibrosis to detect patients’ predisposition to fibrosis [19]. However, efforts are underway to establish a possible treatment. An example is research with the use of mesenchymal stem cells (MSCs). In their paper, Wang et al. demonstrate the potential therapeutic properties of MSCs by reducing collagen deposition and the epithelial–mesenchymal transition process. The authors point to the *TGF-β*/*Smad* signaling pathway as a potential source of process regulation [5]. In another study involving animal models, Venu et al. identified the human nuclear receptor 4A1 (*NR4A1*) as the major regulator of mesenchymal co-cell function and a regulator of myofibroblast function, which consequently may modulate the process of inflammation-related fibrosis [110]. Another promising possible therapeutic agent is the inhibition of IL-36 (interleukin 36) receptor signaling. Scheibe et al., in a study on animal models, observed that when intestinal fibrosis is present in IBD, there are higher levels of IL-36A compared to healthy individuals. Furthermore, they presented that IL-36 increases the expression of genes responsible for regulating fibrogenesis, among other things, so inhibiting IL-36R could reduce intestinal fibrosis. This could be a potential therapeutic option [111]. The authors of other studies have also reached similar conclusions. IL-36 production has been observed in intestinal macrophages, fibroblasts, or intestinal myofibroblasts, where macrophages are one of the sources of intestinal constriction. However, the regulation of IL-36R shows different effects in acute and chronic inflammation. Fibrosis is observed mainly in the presence of chronic inflammation due to the activation of profibrotic cytokines [112,113]. However, the best targeted therapy for intestinal fibrosis remains to be sought.

### 6.2. Potentially Supportive Nutritional Factors

Since macrophages play an important role in the onset of intestinal fibrosis and certain food components can affect macrophage polarization and levels of inflammation, they could also be considered the potential support of diet-based antifibrotic therapy [114]. The *Wnt* pathways regulate homeostasis and, among other things, intestinal fibrosis formation, macrophage-derived *Wnt* ligands are involved, while a probiotic containing *Faecalibacterium prausnitzii* can regulate the expression of the *DACT3* gene, which is related to the *Wnt/JNK* pathway, which consequently can reduce inflammation [115,116]. Marion-Letellier et al., in their paper, indicate that following a high-fat (HF) diet may predispose to organ fibrosis. There is no confirmed effect of the HF diet on intestinal fibrosis, while there are mechanisms that could potentially lead to this condition due to the induction of the epithelial–mesenchymal transition (EMT) and Wnt-β-catenin [117]. Furthermore, the HF diet alters the signaling of peroxisome proliferator-activated receptor-γ (PPAR-γ) signaling, leading to disruption of epithelial integrity, thus altering the composition of the intestinal microbiota, which can trigger inflammation [118]. Another factor that potentially affects the development of intestinal fibrosis is salt. Amamou et al., in their study on animal models, showed that salt, in chronic intestinal inflammation, promotes the onset of intestinal fibrosis by activating intestinal fibroblasts. In addition, it can increase the degree of inflammation [119]. However, curcumin may be a protective factor for the onset of intestinal fibrosis [120,121]. Using animal models, Tao et al. studied the effects of vitamin D on the development of intestinal fibrosis. They observed that vitamin D decreased the production of collagen and ECM in the colon. Furthermore, it decreased *TGF-β1/Smad3* signaling in isolated colonic subepithelial myofibroblasts (SEMFs) [122]. Fermented rice bran can exert antifibrotic effects by modulating *TGF-β* and *Smad3/2*. Furthermore, supplementation decreased inflammatory markers and increased *Clad4*, a component of the intestinal tight junction, among others [123]. Park et al., in turn, studied the effects of *Lactobacillus acidophilus* on levels of inflammation and intestinal fibrosis. They observed that *Lactobacillus acidophilus* reduced type I collagen levels, a marker of activated myofibroblasts, and α-smooth muscle actin [124]. Costa et al. point to the gut microbiome as one of the factors that influence macrophage polarization in both intestinal inflammation and fibrosis. Therefore, a potential protective factor would be an anti-inflammatory diet rich in exogenous antioxidants such as flavonoids, vitamins, and minerals [125,126]. Furthermore, arginine and glutamine show potential antifibrotic effects [117].

However, studies must be designed to confirm the potential therapeutic potential of the diet in intestinal fibrosis due to IBD.

## 7. Conclusions

Although genetic and epigenetic mechanisms have been extensively studied in various biological contexts, there is still a lack of research on intestinal fibrosis, especially in the context of nonspecific inflammatory bowel diseases. Many current analyses are based on preclinical studies. Genetics and epigenetics appear to be crucial in the pathogenesis and progression of IBD. There is a close relationship between genetic variations and fibrosis in Crohn’s disease. Fibrostenotic disease can develop in genetically susceptible individuals and is mainly influenced by interactions with immune and environmental factors. Genotyping and phenotyping studies have shown a correlation between *NOD2* variants and fibrotic stenosis disease. Mutations in genes in the *NOD2* and *ATG16L1* autophagy pathways can contribute to the development of severe fibrosis. However, the mechanisms responsible for *NOD2*-induced fibrosis are still incompletely investigated. On the other hand, epigenetics has a broader scope of action. Regulates physiological and pathophysiological processes by influencing gene expression. Dynamic gene silencing or activation plays a significant role in epigenetic modifications without altering the DNA sequence. Additionally, epigenetics explores the impact of environmental factors on gene expression. These factors include lifestyle, diet patterns, toxins, and oxidative stress. It is important to note that genetic and epigenetic factors interact together in the process of inducing intestinal fibrosis. However, it is crucial to remember that intestinal fibrosis in IBD is a complex process, and its induction and development also depend on other factors, such as environmental and immunological factors. Combining epigenetics with genetics and environmental factors will provide a complete picture for the future of treating and preventing intestinal fibrosis and fibrosis of other organs.

## Figures and Tables

**Table 1:** Summary of genetic and epigenetic factors and their mechanisms in intestinal fibrosis.

Genetic and Epigenetic Factors	Site of Action	Role in Fibrosis
NOD2 (nucleotide-binding oligomerization domain-containing 2)	Is expressed, e.g., in goblet cells, Paneth cells, enterocytes; immune system cells present in the lamina propria; monocytes, macrophages, and dendritic cells [37]	Shifting T lymphocytes toward the production of tissue growth factor beta (TGF-β) cytokines and increasing the deposition of collagen by smooth muscle cells and fibroblasts in the intestine [37] Reduced expression of alpha-defensin in the mucosa [37] Carriers of the 1007fs variant are at risk of intestinal stenosis; the 007fs variant is associated with impaired interleukin-1b production and dendritic cell function [37]
TGF-β (transforming growth factor β)	Can be found in all tissues and can be produced or released by infiltrating cells such as lymphocytes, monocytes/macrophages, and platelets after wounding or inflammation [103]	TGF-β1-directed epithelial–mesenchymal transition [47] Altered Smad3 transduction protein activity and elevated Smad7 levels as a result of impaired TGF-β1 signaling [48] Induction of myofibroblast accumulation by promoting EMT and Endo-MT [47] Increasing the proliferation of myofibroblasts and making them resistant to apoptosis [47] Myofibroblasts promote fibrosis by inducing collagen and MMP under TGF-β simulation [47] ECM remodeling by increasing TIMP expression [47]
TLRs (toll-like receptors)	Are expressed in myeloid cells [37]	Increased expression of TLR4 in cells of the ileal crypt [50]
Il23R (interleukin 23 receptor)	Is highly expressed on the cell membrane of memory T cells and other immune cells such as monocytes, natural killer cells, and dendritic cells [37]	The association of *IL23R* variants with CD and UC inflammation was observed [37]
ATG16L1 (autophagy-related 16-like 1)	Is expressed in intestinal epithelial cells, as well as in macrophages and leukocytes [104]	Impairment of autophagic function and anti-inflammatory activity (possibly reduced ability to generate a specific type of macrophage (Mφind) [37] Stimulation of mesenchymal cells to produce large amounts of collagen and other fibrogenic particles [37] Changing the reactions of immune cells to bacterial components [37] Variant *T300a* increases the production of cytokines driven by *NOD2* [37]
DNA (deoxyribonucleic acid) methylation	It occurs primarily occurs on CpG dinucleotides, with most CpGs concentrated in CpG islands [73]	Specific gene hypermethylation and general methylation alterations have been observed in organs during IBD [75,76,77] Differentially methylated position (DMP) (*cg16176675*) was found to be significantly hypermethylated in patients with IBD [81] DNA methylation abnormalities in the intestinal mucosa were identified in the *HLA-DRB1*, *YPEL5*, and *CBLB* genes in CD patients [82,83]
Histone modifications	Histones	*H3K27me3* can potentially control the outer follicles for the regulation of Th17 in patients with UC [91] The silencing of *SETD8* regulates the expression of the p62 protein, thereby blocking the inflammatory response of the colon. *SETD8* could potentially be used as a therapy for patients with IBD in the future [92] Protein arginine methyltransferase (PRMT) exhibits [94]
RNA (ribonucleic acid) interference	RNA	Increased expression of miR-21 in muscle cells and myofibroblasts in CD patients leads to continuous activation of TGF-β1 signaling, resulting in excessive production of collagen and extracellular matrix, leading to fibrosis [101] The Wnt-β-catenin signaling pathway is activated during intestinal fibrosis [102]

## Data Availability

Not applicable.
